# Atypical metanephric adenoma: Shares similar histopathological features and molecular changes of metanephric adenoma and epithelial-predominant Wilms’ tumor

**DOI:** 10.3389/fonc.2022.1020456

**Published:** 2022-10-14

**Authors:** Xiaoxue Yin, Xingming Zhang, Xiuyi Pan, Junya Tan, Linmao Zheng, Qiao Zhou, Ni Chen

**Affiliations:** ^1^ Department of Pathology, Laboratory of Pathology, West China Hospital, West China Medical School, Sichuan University, Chengdu, China; ^2^ Department of Urology, West China Hospital, West China Medical School, Sichuan University, Chengdu, China

**Keywords:** metanephric adenoma, atypical metanephric adenoma, Wilms’ tumor, clinicopathological features, *BRAF* V600 mutation

## Abstract

**Background:**

Metanephric adenomas (MAs) are rare, benign renal tumors. Wilms’ tumors (WTs) are malignant embryonic tumors that originated from nephrogenic blastemal cells. However, some tumors have similar morphology to both MA and epithelial-predominant WT, which makes differential diagnosis difficult. We aimed to analyze the morphological, immunophenotypic and molecular changes in overlapping cases to explore their attribution.

**Methods and results:**

Twenty MAs, ten WTs, and nine cases with MA/WT overlapping histological features were studied. Twenty tumors demonstrated the typical morphological spectrum of MA with high cellularity and were composed of tightly packed small, uniform, round acini with a lower Ki67 index. Almost all MAs (94.7%, 18/19) were detected with *BRAF* V600E mutation. The ten WTs were epithelial-predominant WTs with glands, rosettes and glomerular structures, which also showed a higher Ki-67 index (up to 60%), invasive growth patterns, and a lack of *BRAF* mutation. However, the other nine overlapping cases showed two components: typical MA-like areas and epithelial WT-like areas. The cells of the WT-like areas were tubular, columnar and showed marked cytological atypia, with a Ki-67 proliferative index of up to 30%. The immunophenotype of these overlapping lesions was not significantly different from that of typical MA and they positively expressed WT1 and CD57. The *BRAF* V600E mutation was detected in both WT-like and MA-like areas in nine overlapping tumors. The follow-up data of 31 patients were analyzed, with a median follow-up time of 66 months (range, 8-45 months). Even though most patients with WT underwent radiotherapy or chemotherapy after surgery, two died, and one had liver metastasis. No MA or overlapping cases showed any evidence of recurrence or metastasis after surgery.

**Conclusions:**

The molecular changes in tumors with overlapping morphological features were the same as those of typical MA; thus, we think that these tumors should be classified as MA and further called atypical MA. It is important to note that atypical MA is not a neglected subtype of MA. It possesses different histological morphology and a higher Ki-67 index but has the common imaging characteristics, immunophenotype and gene expression as typical MA, and patients usually have a good prognosis.

## Introduction

Metanephric adenoma (MA) is an uncommon kidney neoplasm that accounts for 0.2-0.7% of primary renal epithelial tumors (1). It is often asymptomatic, generally occurs in adults and has a significant predominance in females (2, 3). Most MAs feature a small solid, well-circumscribed, unilateral renal mass composed of primitive metanephric tubular cells and can be diagnosed by routine hematoxylin and eosin staining. Nevertheless, some MAs may also exhibit atypical morphology or overlap with other tumors. MA should be differentially diagnosed as the solid variant of papillary renal cell carcinoma (PRCC), epithelial-predominant Wilms’ tumor (WT), and mucinous tubular and spindle cell carcinoma (4, 5). MA can also be challenging to diagnose due to its confusing histopathological morphology.

WT, also known as nephroblastoma, is another tumor that can form primitive renal tubules. In contrast to MA, WT has a younger onset age and is the most common embryonal tumor in children (6). Tumors generally mimic the cell types observed during normal nephrogenesis, with the classical triphasic WT comprising undifferentiated blastemal cells with differentiation toward both stromal and epithelial elements (7). Epithelial-predominant WT is a rare subtype of WT, that belongs to intermediate-risk tumors, and more than 67% of the tumor cells are epithelial structures (8). The epithelial components of tumors are usually rosette-like but may also be tubular or papillary, with or without heterologous epithelial differentiation. Degeneration, liquefaction, necrosis, hemorrhage, and metastasis are common in embryonal tumors.

It is worth noting that some renal tumors have overlapping morphologic features of epithelial-predominant WT and MA. Overlapping lesions were first reported in 1995, and the author occasionally found some epithelial renal tumors with features of epithelial WT, however, the cells had a bland and adenomatous appearance with focal areas similar to MA (9). Subsequently, more cases with overlapping features have been reported, which were described as either typical MA with active mitotic activity or MA with a morphology similar to epithelial WT (5, 10). However, in addition to morphological descriptions, the diagnosis and attribution of these cases remain unclear.

In this study, we summarized a series of MA/WT overlapping tumors and compared their morphology, Ki-67 index, *BRAF* mutation status, and prognosis with typical MA and epithelial WT, with the aim of highlighting and further clarifying the attribution of these tumors.

## Materials and methods

### Patients and samples

We reviewed MAs and epithelial-predominant WTs diagnosed at the West China Hospital of Sichuan University from 2008 to 2021. Formalin-fixed paraffin-embedded (FFPE) blocks were retrieved, and the corresponding slides from all cases were re-reviewed independently by two genitourinary pathologists (YXX, CN). Finally, 39 tumors were studied: 20 typical MAs, 9 renal tumors with overlapping morphological features of epithelial-predominant WT and MA, and 10 other cases of epithelial-predominant WT were selected as controls.

### Immunohistochemistry (IHC)

IHC staining was performed on 4-μm-thick formalin-fixed paraffin embedded tissue sections by using the following antibodies: WT1 (6F-H2, DAKO, 1:100), CD57 (NK-1, Zhongshan Golden Bridge, 1:100), CD56 (123C3, Zhongshan Golden Bridge, 1:100), EMA (M0613, DAKO, 1:100), CK7 (OV-TL12/30, Zhongshan Golden Bridge, 1:100), and Ki-67 (MIB-1, Maixin, 1:100). All immunohistochemical staining was performed using the Roche BenchMark ULTRA automated staining system (Roche, Basel, Switzerland) according to the manufacturer’s protocols. Ki-67 was assessed as follows: five random fields of tumor sections were randomly selected to calculate the average proportion of positive cells under a magnification of ×400, and the Ki-67 index was presented as a percentage.

### Detection of *BRAF* V600 mutation by polymerase chain reaction (PCR)

An FFPE DNA Kit (Qiagen, Hilden, Germany) was used to extract DNA from FFPE tissue samples from the tumor according to the manufacturer’s protocol. Polymerase chain reaction (PCR) experiments were carried out with Taq HS (TaKaRa, Shiga, Japan), forward primer (5’-TCATAATGCTTGCTCTGATAGGA-3’) and reverse primer (5’- GCCAAAAATTTAATCAGTGG A-3’) primers. The PCR conditions were as follows: 94°C for 3 minutes (min); 35 cycles of denaturation at 94°C for 1 min, annealing at 60°C for 50 seconds (s), and extension at 72°C for 90 s; and a final extension at 72°C for 10 min. The amplified fragments were resolved by agarose gel electrophoresis, recovered by gel extraction (Qiagen, Hilden, Germany), and sequenced. For cases with overlapping morphology of WT and MA, manual microdissection was performed to distinguish WT-like and MA-like areas, DNA was then extracted from different areas for further PCR and sequencing.

## Results

### Typical MA (N = 20)

Twenty cases of typical MA (case 1-20) are listed in **Table 1**, involving 14 females and 6 males, and the age at diagnosis ranged from 3 to 70 years (median = 39 years). Most MA patients had no symptoms and were usually discovered by physical examination, while some patients had gross hematuria, bellyache, and abdominal mass as their primary clinical features. All patients had unilateral renal masses, and MA appeared to be more in the right kidney (left: right = 7: 13). Computer tomography scan showed a renal round exophytic soft tissue dense mass with a clear boundary, with or without a capsule. The mass density on the unenhanced scan was equal to or slightly higher than that on the kidney, which has uniform density. On enhanced scan, the mass density changed gradually mild to moderately uneven.

**Table 1:** Cases in this study.

Case	Age(Y)\Sex	Tumor Size(cm)	Tumor Morphology (%)	Treatment	Ki-67 (%)	WT 1	CD 57	CD 56	EMA	CK 7	*BRAF* Status	Follow up(mo)	Status
1	26/F	4.0	MA	NA	2	+	+	NA	–	–	V600E	NA	NA
2	28/F	7.5	MA	NSS	5	+	+	NA	–	–	V600E	143	NED
3	49/F	3.0	MA	NSS	NA	+	+	–	–	–	NA	126	NED
4	59/M	7.0	MA	RN	1	+	+	–	–	–	V600E	105	NED
5	14/M	18.0	MA	RN	5	+	+	–	–	–	V600E	103	NED
6	27/F	5.5	MA	RN	5	+	+	+	–	–	V600E	111	NED
7	50/F	4.5	MA	RN	1	+	+	+	NA	–	V600E	96	NED
8	52/M	15.0	MA	RN	3	NA	NA	NA	NA	NA	V600E	74	NED
9	60/F	2.5	MA	NSS	3	+	+	NA	–	–	V600E	69	NED
10	30/F	9.0	MA	NA	NA	+	+	+	–	–	V600E	66	NED
11	20/F	NA	MA	NA	5	+	+	+	–	NA	V600E	NA	NA
12	28/F	3.5	MA	NSS	15	+	+	+	NA	–	V600E	60	NED
13	58/M	3.5	MA	RN	5	+	+	+	–	–	V600E	59	NED
14	3/M	5.5	MA	RN	2	+	+	+	NA	–	V600E	56	NED
15	48/F	4.7	MA	NSS	2	+	+	–	–	–	V600E	53	NED
16	70/M	4.0	MA	NSS	3	+	+	+	NA	–	V600E	NA	NA
17	36/F	4.5	MA	RN	15	+	+	–	–	–	–	35	NED
18	33/F	1.5	MA	NSS	5	+	+	NA	NA	–	V600E	24	NED
19	42/F	2.5	MA	NSS	10	+	+	NA	–	–	V600E	12	NED
20	65/F	4.0	MA	NSS	5	+	+	–	–	–	V600E	NA	NA
21	17/M	3.0	WT	NSS	25	–	–	NA	–	–	–	8	**Dead**
22	13/M	2.5	WT	RN + chemotherapy +radiotherapy	30	–	–	+	–	–	–	53	**NED, liver metastasis occurred 2 years after surgery**
23	38/F	4.5	WT	RN + chemotherapy + radiotherapy	40	+	NA	+	+	–	–	41	NED
24	3/M	10.0	WT	RN	60	–	NA	NA	–	+	–	NA	NA
25	5/F	12.0	WT	RN	40	+	NA	NA	NA	NA	–	76	NED
26	3/F	14.0	WT	RN + radiotherapy	25	+	NA	+	–	NA	–	17	**Dead**
27	5/M	10.0	WT	RN + chemotherapy	NA	+	NA	+	NA	NA	–	70	NED
28	1/F	8.0	WT	RN	30	+	NA	NA	+	+	–	81	NED
29	5/F	5.0	WT	RN	40	–	–	+	–	NA	–	81	NED
30	1/M	4.5	WT	RN + chemotherapy	30	+	+	+	+	NA	–	80	NED
31	21/M	6.0	MA-like(95) + WT-like(5)	RN	MA-like(2) + WT-like(5)	+	NA	NA	NA	NA	MA-like: V600E, WT-like: V600E	145	NED
32	20/F	NA	MA-like(60)+WT-like(40)	NA	MA-like(1) + WT-like(10)	+	NA	NA	–	–	MA-like: V600E, WT-like: V600E	NA	NA
33	20/F	4.5	MA-like(60)+WT-like(40)	NSS	MA-like(2) + WT-like(10)	+	+	+	–	NA	MA-like: V600E, WT-like: V600E	66	NED
34	14/F	3.7	MA-like(40)+WT-like(60)	NSS	NA	+	+	NA	–	NA	MA-like: V600E, WT-like: V600E	NA	NA
35	31/F	5.0	MA-like(30)+WT-like(70)	RN	MA-like(1) + WT-like(15)	+	+	+	–	–	MA-like: V600E, WT-like: V600E	130	NED
36	31/M	4.8	MA-like(20)+WT-like(80)	RN	MA-like(2) + WT-like(10)	+	+	+	–	–	MA-like: V600E, WT-like: V600E	NA	NA
37	37/F	2.0	MA-like(20)+WT-like(80)	RN	MA-like(2) + WT-like(30)	+	NA	NA	–	NA	MA-like: V600E, WT-like: V600E	20	NED
38	38/F	6.1	MA-like(10)+WT-like(90)	NSS	MA-like(5) + WT-like(20)	+	+	NA	–	–	MA-like: V600E, WT-like: V600E	46	NED
39	51/M	3.0	MA-like(5)+WT-like(95)	RN	MA-like(5) + WT-like(30)	+	+	NA	–	NA	MA-like: V600E, WT-like: V600E	35	NED

Y, year; F, female; M, male; MA, metanephric adenoma; WT, Wilms’ tumor, NSS, nephron-sparing surgery; RN, radical nephrectomy, mo, month; NA, not available; NED, no evidence of disease.

**Figure 1:**
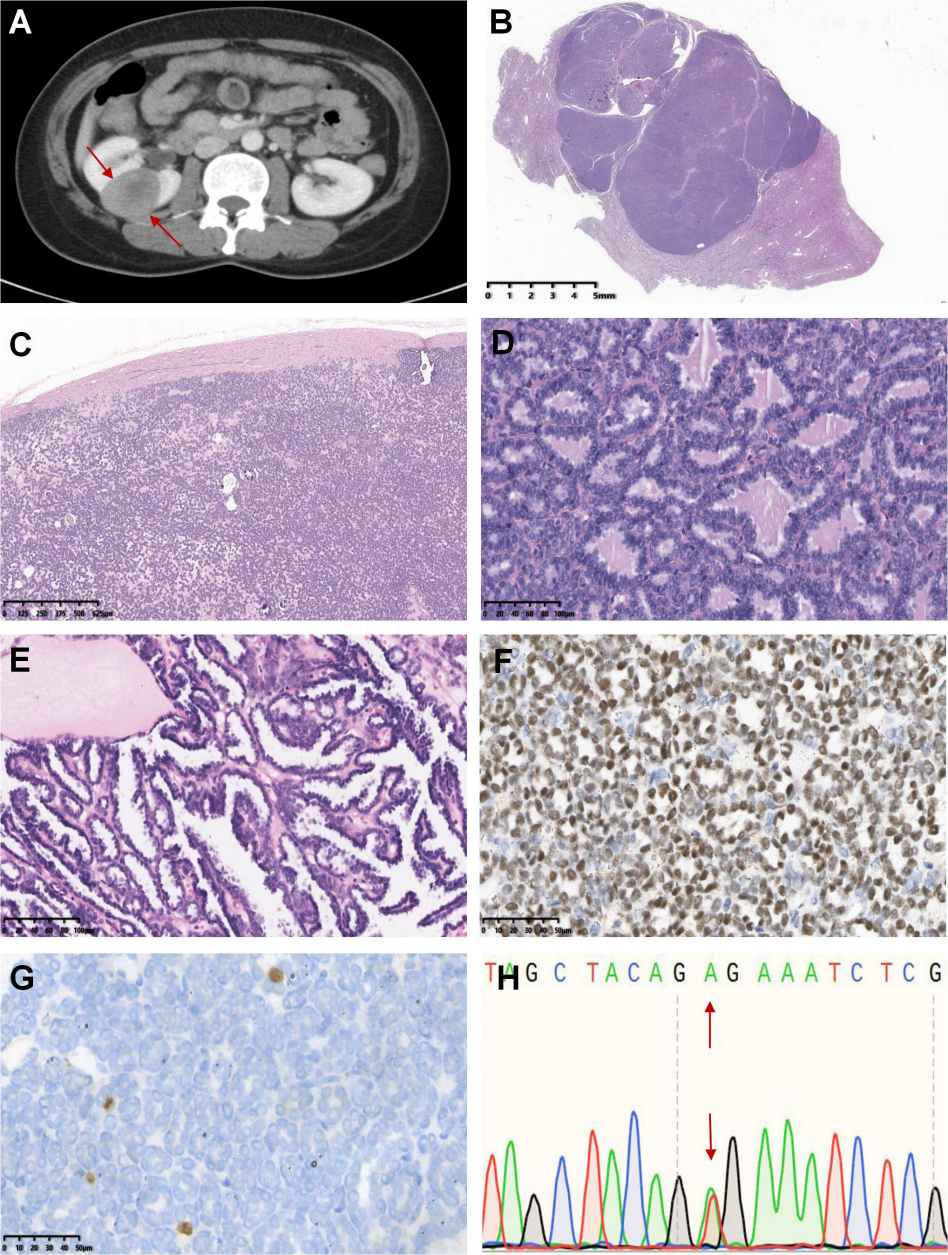
Case 1-20 demonstrated the typical histomorphological spectrum of MA. The representative morphological images were shown below: Axial computer tomography scan displayed a soft tissue mass of 3.1 cm × 3.7 cm in the upper pole of the right kidney, with a clear boundary and obvious enhancement (case 2, **A**). The tumor was multinodular, well-defined and usually abutted directly against the normal renal tissue without a pseudocapsule (case 18, **B**). Psammoma bodies were common and may be numerous, and the stroma ranged from inconspicuous to loose and edematous (case 16, **C**). Tumors were highly cellular and composed of crowded small, round acini, papillae or antler tubular structures, and mitotic figures were rare or absent (case 19 and 7, **D, E**). Tumor cells showed diffusely positive staining for WT1 (case 7, **F**) but negative expression or only a few cells expressed Ki-67 (case 7, **G**). Sanger sequencing revealed that the tumor harbored the T1799A mutation in exon 15 of the *BRAF* gene (V600E mutation) (case 7, **H**).

Macroscopically, most tumors were nodular, including one solid-cystic tumor and two tumors with macroscopic necrosis. The maximum tumor diameter ranged from 1.5 to 18.0 cm (median, 4.5 cm). Histologically, these tumors were well defined and usually abutted directly against normal renal tissue without a pseudocapsule. All 20 cases were highly cellular and composed of crowded small, round acini, tubular, glandular, antler tubular, papillary, and solid patterns, along with glomeruloid bodies. Tumor cells were bland, uniform, round, or oval with scant cytoplasm, monomorphic nuclei, fine chromatin, and small and inconspicuous nucleoli. The stroma ranged from inconspicuous to loose and edematous. Hyaline degeneration and psammoma bodies were common and might be numerous. Mitotic figures were rare or absent. In addition, all 19 cases were tested positive for WT1 and CD57 expression by IHC, and the Ki-67 index ranged from 1% to 15%. Sanger sequencing showed that 18 of these 19 (94.7%) cases had the *BRAF* V600E mutation. The 1799 base in exon 15 of the *BRAF* gene was changed from T to A, leading to the related valine being replaced by glutamic acid (**Figure 1**).

All patients received nephron-sparing surgery or radical nephrectomy without postoperative adjuvant therapy, including targeted therapy, chemotherapy or radiotherapy after surgery. The follow-up lasted anywhere from 12 to 143 months (mean = 74.5 months, median = 67.5 months), and no patients showed signs of a recurrence or metastasis.

### WT (N = 10)

Ten epithelial-predominant WTs (case 21-30), with more than 67% of the tumor cells being epithelial structures, were enrolled as the control group. The patients included 5 males and 5 females with ages ranging from 1 to 38 years (median = 5 years). An abdominal mass was the most common symptom in patients, and computer tomography scan revealed a large lesion in the kidney. In contrast to the crescent-like enhancement of the residual renal parenchyma, the tumors were heterogeneous in density with mild to moderate uneven enhancement. Some tumors demonstrated pushy growth and infiltrated the peripheral blood vessels and nerve tissue.

**Figure 2:**
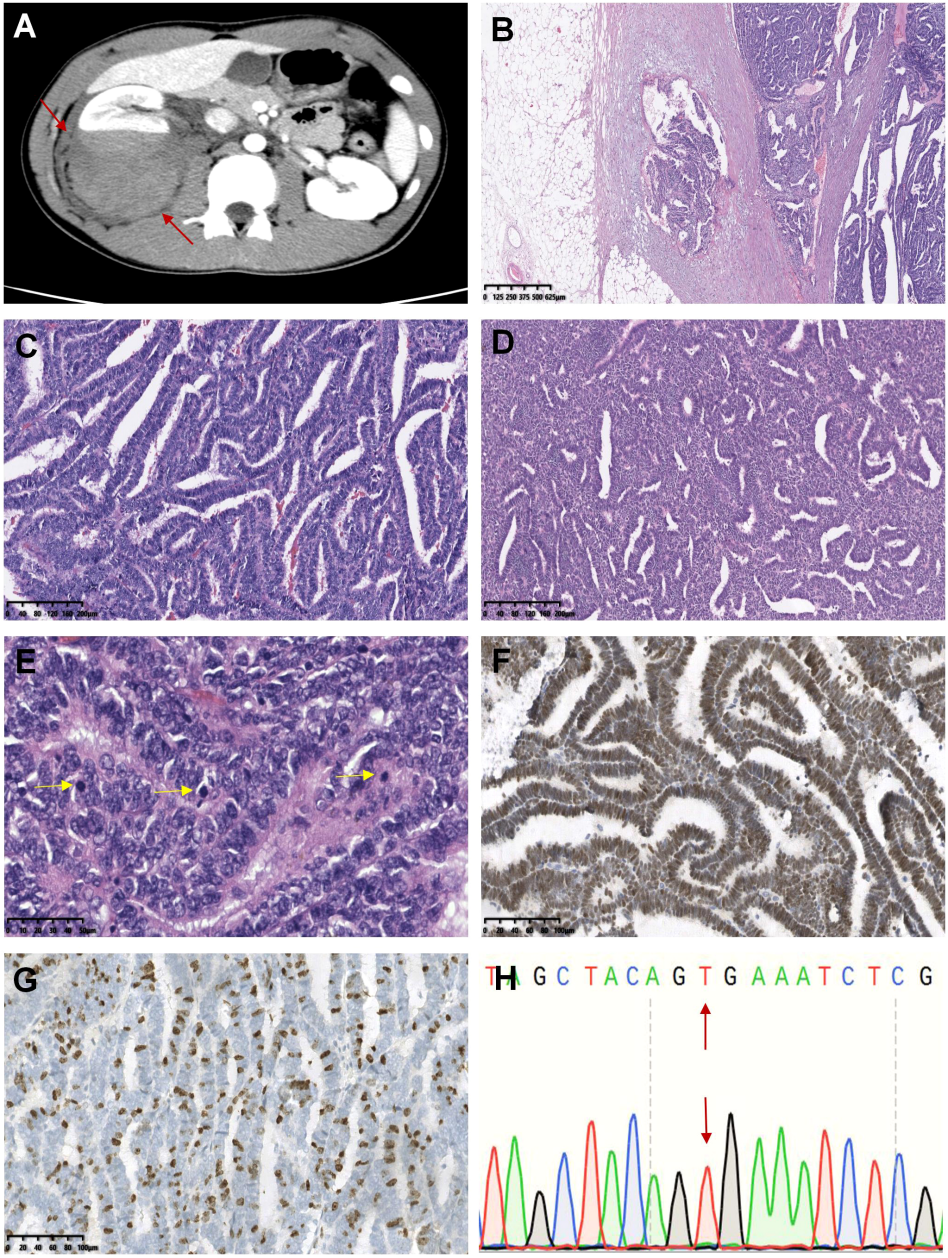
Case 21-30 were epithelial-predominant WT. The representative histomorphological images were as follows: In the axial computer tomography scan, the patient’s right kidney was characterized by a large swollen mass with uneven density and a clear boundary with the surrounding renal parenchyma, which appeared to invade the perirenal tissue (case 22, **A**). Microscopically, the tumor invaded the renal capsule, and carcinoma was observed in the renal fibrous membrane (case 22, **B**). Tumor cells were arranged in obvious papillary, acinar or duct structures (case 22 and 23, **C, D**). Pathological mitotic figures were easily seen and were indicated by the yellow arrows (case 22, **E**). WT1 (case 22, **F**) and Ki-67 (case 22, **G**) were highly expressed in tumors. No *BRAF* V600E mutation was observed (case 22, **H**).

All patients underwent radical nephrectomy, and half of them were treated with vincristine-based therapy or radiotherapy after surgery. Most lesions were accompanied by hemorrhage, necrosis, calcification, or cystic degeneration due to excessive growth. These tumors showed epithelial differentiation and were composed of tubules, rosettes, and primitive glomeruli. The tumor cells were more heteromorphic, with active mitosis and more pathological mitosis. The Ki-67 index was usually ≥ 30%, and WT1 was positively expressed in 60% (6/10) of tumors. *BRAF* mutations were performed, but none of these lesions were detected with the V600 mutation (**Figure 2**). By the end of follow-up, nine patients were contacted, and the follow-up lasted anywhere from 8 to 81 months (mean = 56.3 months, median = 70 months). Unfortunately, two patients died, and one patient developed liver metastases two years after the surgery.

### Renal tumors with overlapping morphological features of MA and epithelial-predominant WT (N = 9)

Nine tumors (case 31-39) shared the overlapping morphological features of epithelial-predominant WT and MA. The patients included 6 females and 3 males, with a median age of 31 years (range, 14-51 years). Most tumors showed heterogeneous-enhancement on computer tomography scans, and the imaging features were similar to those of typical MA. The tumors were nodular or multinodular, well-circumscribed, and expanded in size.

**Figure 3:**
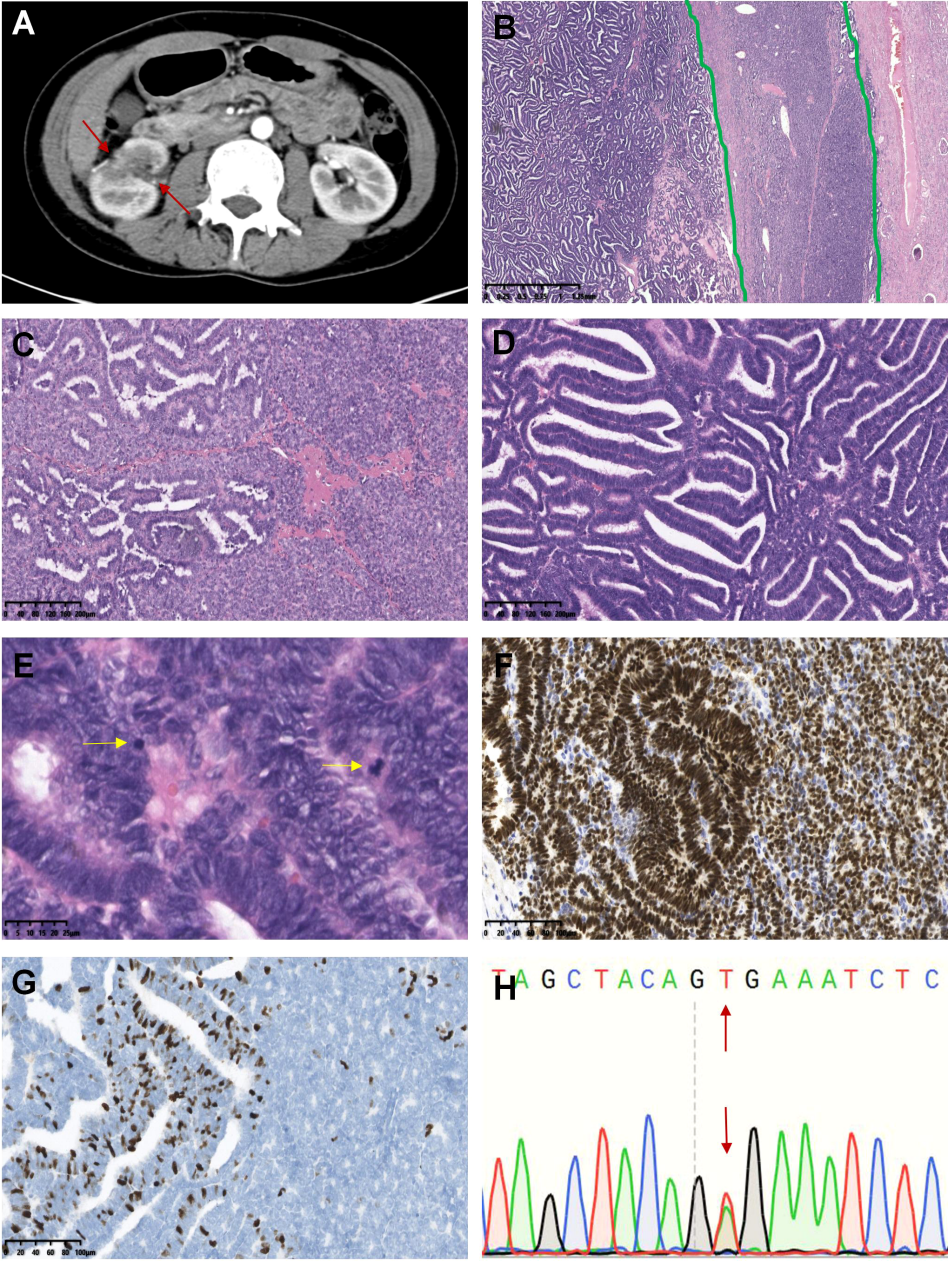
Tumors with overlapping morphologic features of MA and epithelial-predominant WT (case 31-39). The representative morphological images were displayed as follows: A large mass was observed in the middle and upper parts of the right kidney in computer tomography scan (axial, case 38). The mass was irregularly bound, compressing the surrounding renal tissue, and the tumor parenchyma was markedly heterogeneously enhanced **(A)**. The green lines divided the tissue into three areas: the tumor demonstrated discrete areas that were predominantly composed of epithelial WT (left) but had a narrow strip area (middle) associated with cuboidal mitotically inactive epithelium consistent with MA **(B)**. Excessive abruptness and unclear boundaries between the two structures of the tumor **(C)**. The majority of tumors demonstrated primitive columnar-shaped neoplastic cells with papillotubular or rosette-like architectures, typical of epithelial-predominant WT **(D)**. The yellow arrow indicates pathological mitotic figures **(E)**. WT1 was positively expressed in the tumor **(F)**, and Ki-67 expression in the WT-like area was significantly higher than that in the MA-like area **(G)**. The tumor showed the *BRAF* V600E mutation **(H)**.

Both typical MA-like areas and primitive epithelial components (WT-like areas) were observed in these lesions, and epithelial WT-like areas accounted for 5-95% of the neoplasms in different cases. The tumor cells demonstrated marked cytological atypia; some of them were oval or polygonal and tightly packed into the solid and nested structures, while others were cubic or highly columnar and showed different epithelial differentiation into tubules (with earlier forms resembling rosettes), papillae, glands, cystic degeneration with fine chromatin, high nucleocytoplasmic ratio, scant and pale or light pink cytoplasm, and obvious mitotic activity. Calcification and psammoma bodies were easily observed. Notably, we observed significant hyaline degeneration and calcification in the tumor stroma, forming a thick pseudocapsule (case 36). The immunohistochemistry staining results are listed in **Table 1**. All tumors expressed WT1 (9/9) and CD57 (6/6). The Ki-67 index was significantly higher in the epithelial WT-like area (5-30%) than in the MA-like area (1-5%) for the nine tumors. *BRAF* mutation analysis was performed separately on tissues from both MA-like and WT-like areas and yielded identical V600E mutation (**Figure 3**).

Among them, two cases were almost completely composed of papillotubular architecture in the primitive epithelial components, and the WT-like areas accounted for more than 90% of the tumor. The first case (case 38) was a 31-year-old woman who presented with a 5.0 cm renal mass that was partially covered by a membrane on the surface. Necrosis, hemorrhage, and hyalinized stroma were observed in the lesion. The tumor demonstrated discrete areas that were predominantly composed of epithelial WT and had a narrow strip area associated with cuboidal mitotically inactive epithelium consistent with MA. Tumor cells in the WT-like area were highly-columnar, with crowded high-columnar nuclei and active mitotic features. The second case (case 39) was a 51-year-old man with a 3.0 cm renal neoplasm. The tumor was multinodular and invaded the perirenal fat of the kidney, which was morphologically consistent with epithelial WT in most areas, and MA-like epithelium in a few areas. The tumor was well demarcated from the surrounding renal tissue, but the demarcation between the WT-like and MA-like areas was unclear.

**Figure 4:**
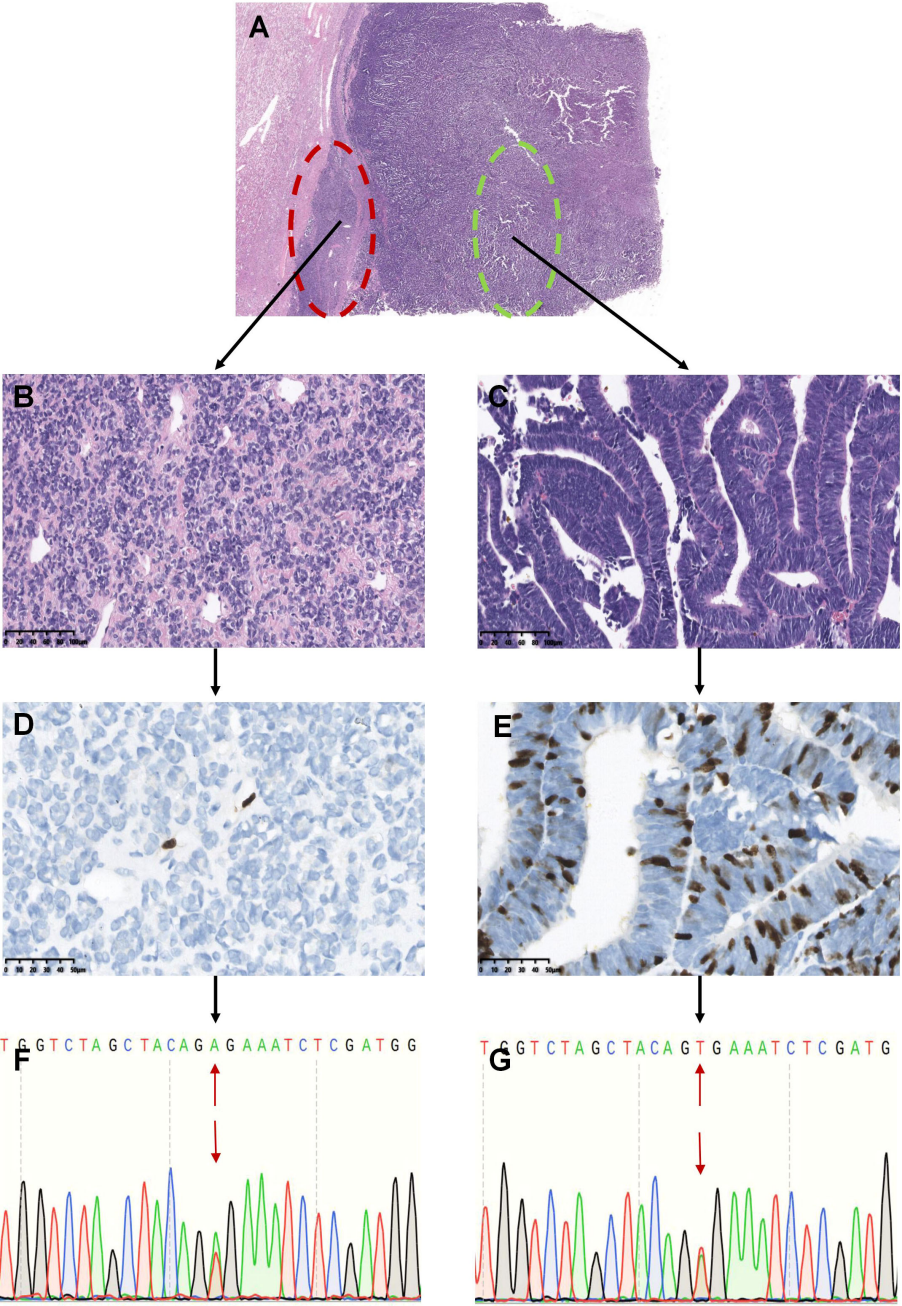
Analysis of *BRAF* mutations in different areas of renal tumors with overlapping morphology (Represented by case 37). The tumors showed overlapping morphological features of epithelial-predominant WT and MA **(A)**. The red circle indicates typical MA-like features with mild cells and hyalinized stroma, and the tumor cells were oval or polygonal and tightly packed in the solid and nested areas **(B)**. The green circle shows that the tumor consisted of primitive epithelial components with a papillotubular architecture, similar to epithelial WT. The tumor cells were tall columnar, with fine chromatin, a high nucleocytoplasmic ratio, crowded high columnar nuclei, and minimal mitotic activity **(C)**. Only a few cells expressed Ki-67 in the typical MA-like areas **(D)**, whereas more Ki-67 was expressed in the epithelial WT-like areas **(E)**. Tumor cells from both areas showed the *BRAF* V600E mutation **(F, G)**.

In summary, these overlapping lesions in the 9 cases showed different proportions of MA-like and WT-like areas, with positive expression of WT1 in both areas. The Ki-67 index was lower in the MA-like area and higher in the epithelial-predominant WT-like area, reflecting the different mitotic activities within the lesions. However, *BRAF* V600E mutations coexisted in both morphological areas of the tumor (**Figure 4**). At the end of follow-up, no recurrence or metastasis was observed in the 6 patients with follow-up data (ranging from 20-145 months, mean = 73.6 months, median = 56 months). Therefore, based on morphological features, immunomarkers, molecular changes, and prognosis, we believe that these 9 overlapping lesions were more similar to MA and could be called atypical MA.

## Discussion

MA is a rare renal tumor characterized by the proliferation of small epithelial cells and is classified as a benign renal epithelial tumor. WT is a malignant embryonic tumor derived from renal blastemal cells. However, in cases with overlapping morphological features of WT and MA, it is difficult to diagnose (4). In this study, we summarized and analyzed a group of cases, including typical MAs, epithelial-predominant WTs and renal tumors with overlapping morphological features of MA and WT (**Table 2**). Based on the comparison of their clinicopathological features and molecular changes, we found that cases with WT/MA overlapping features were closer to MA due to the same immunophenotype, molecular changes, and clinical outcomes, while these cases had different morphologies and a higher Ki-67 index. Therefore, we classified these overlapping cases as MA and named them atypical MA.

**Table 2:** The histological criteria used to classify tumors into three groups.

	Border	Tumor parenchymal cells	Tumor stromal cells	Secondary changes
**MA**	The junction with the kidney is usually abrupt and without a pseudocapsule.	The tumor is typically highly cellular and consist of densely arranged small, uniform vesicles or tubules. The tubules can show branching and intraluminal tufts, producing glomeruloid structures with small cuboidal cells of uniform size, scanty cytoplasm, round or ovoid nuclei, fine nuclear chromatin, inconspicuous nucleoli, and rare or absent mitotic figures.	The stroma ranges from inconspicuous to loose and oedematous, with no obvious vascular structure.	Hyaline degeneration, calcifications and psammoma bodies are common, with hemorrhages and cystic changes visible.
**Epithelial-predominant WT**	Typical circumscribed, encapsulated, pushing border.	The viable tumor consists of at least 66% of epithelial structures. The tumor cells are arranged in tubular, vesicular or papillary structures with short or high columnar nuclei perpendicular to the basement membrane, marked nuclear atypia, coarse nuclear chromatin and more mitotic figures.	The mesenchymal component is diverse and differentiation of smooth muscle, striated muscle, fibroblasts, adipose tissue, cartilage and bone can be seen.	Tumors are often associated with hemorrhage, necrosis, calcification, etc.
**Atypical MA**	With or without a pseudocapsule.	Tumors share the overlapping morphologic features of epithelial-predominant WT and MA, both typical MA-like and WT-like areas were observed in these lesions.		

MA, metanephric adenoma; WT, Wilms’ tumor.

To date, more than 300 cases of MA have been reported (1–3, 10–13). The patients ranged from children to elderly individuals, with a median age of approximately 50 years, with an obvious female preponderance (female/male = 1/2-1/3) (10, 11, 14). The most common symptoms are pyrexia, hematuria, lumbar pain and an abdominal mass; however, most patients have no symptoms and are found incidentally (3). In our study, the ages of 20 patients with typical MA were younger than those of most previously reported cases, ranging from 3 to 70 years (median = 39 years), with a significant female tendency (male-to-female ratio, 6:14). However, the median age of 9 atypical MAs was 31 years, with no significant difference from the typical MAs, but much older than the epithelial-predominant WT (median = 5 years). Overall, atypical MA is more similar to typical MA in terms of age at onset.

Macroscopically, MA is usually a nodular mass of various sizes, but multifocality and cystic degeneration are rare (15–18). In the present study, 9/29 tumors showed multifocality, and 3 tumors showed cystic degeneration. Histologically, all 9 atypical MAs were composed of different proportions of MA-like and WT-like structures. Epithelial WT-like areas accounted for 5%-95% of the tumors. Among them, two tumors were composed of papillotubular architecture (more than 90% of the tumor area) with a high nucleocytoplasmic ratio, active mitotic features, and significant necrosis. These 9 cases showed different levels of tumor cytological atypia, and it was difficult to differentiate them from WT based on cellular morphological features. In fact, many tumors with similar morphology of epithelial components that need to be distinguished from MA, especially the solid variant of PRCC and epithelial-predominant WT (17).

Some uncommon histological morphologies of MA still exist. The first case of MA with an atypical morphology was reported in 1995 (19). In 2007, Jain et al. proposed the concept of atypical MA. A case of MA with atypical histological features characterized by various-sized nuclei, hyperchromasia, prominent nucleoli, and approximately 2/10 high-power fields of mitotic activity was observed in the cellular areas (20). Subsequently, “malignant MA” was proposed, which was considered to comprise hypercellular uniform cells in a solid-acini pattern, and the cells varied in size with small uniform nuclei, prominent nucleoli and with or without increased numbers of mitoses (12). Wobker et al. reported a group of cases that morphologically overlapped MA and WT, which were divided into typical MAs with unusually prominent mitotic activity, and epithelial WTs with areas resembling MA (4). In addition to WT, composite tumors of MA with other malignant components have also been reported (12, 21–26). However, no consensus has been established on the attribution and nomenclature of these tumors, and the term “malignant MA” remains controversial.

Due to the overexpression of WT1 and CD57 in both MA and WT, IHC is less useful for differential diagnosis. In the present study, WT1 and CD57 were positively expressed in 19 typical MAs, whereas 6/10 and 1/3 of the epithelial-predominant WTs expressed WT1 and CD57, respectively. In addition, Ki-67 indices ranged from 1% to 15% in typical MAs and 25% to 60% in WTs, with significant differences between the two tumors. For the 9 renal tumors with overlapping morphologic features of MA and WT, WT1 and CD57 were positively expressed in both MA-like areas and epithelial WT-like areas, and Ki-67 indices of MA-like areas were 1-5%, less than that in epithelial WT-like areas (5-30%). These results suggest that the cell proliferative activity and immunophenotype of tumors with overlapping morphology were between those of typical MA and WT, and that they were closer to MA.

Molecular analyses play an important role in the differential diagnosis of renal epithelial neoplasms. The *BRAF* V600E mutation has been found in 66.7% to 100% of MA tumors, and the V600D missense mutation, V600K mutation, and compound V600D and K601L missense mutations have recently been reported (2, 11, 27–30). However, only two molecular studies have reported on tumors with overlapping WT/MA morphological features, especially *BRAF* mutations. One article reported 9 overlapping cases, 4 of which showed a *BRAF* V600E mutation in both epithelial WT-like and MA-like areas (4). Another study found that *BRAF* mutations were of diagnostic interest in overlapping lesions because *BRAF* mutations were detected only in typical MA and overlapping cases, but did not exist in epithelial WT (31).

In the present study, we performed manual microdissection of the tissue to distinguish WT-like areas and MA-like areas for overlapping lesions and further separately detected the *BRAF* mutation status. Finally, both WT-like areas and MA-like areas showed that the 1799 base in exon 15 of the *BRAF* gene changed from T to A, and only the *BRAF* V600E mutation was detected in our cases. In total, 94.8% (18/19) of the typical MAs and 100% (9/9) of the atypical MAs showed this genetic change. The absence of other *BRAF* mutations in our cases may be due to ethnic differences in the Chinese population. Moreover, *BRAF* V600E mutations in common non-MA renal tumors were either extremely infrequent (less than 1%) or absent. *BRAF* mutations were not found in our WT cases. To date, only a few epithelial WTs have been found to harbor this mutation (4, 11, 28, 31, 32). Therefore, *BRAF* mutation detection is helpful in differentiating MA from epithelial-predominant WT cases.

Currently, most MAs have a good prognosis. However, the ability of MA to become malignant has also been reported. Some studies have found that a small subset of these tumors have atypical histological characteristics, an exponential growth pattern (12) or even coexist with other malignant tumors (21, 24, 33). The duration of follow-up for our cases (including typical and atypical MA) ranged from 12 to 145 months, and none of them showed any evidence of recurrence or metastasis. Therefore, we tend to consider MA to be an indolent tumor, and cases 31-39 in our study are more suitable to temporarily named atypical MA rather than malignant MA or epithelial-predominant WT resembling MA with the *BRAF* V600E mutation.

## Conclusion

In this study, we reported 9 atypical MAs that were younger than most reported patients, and all cases harbored the *BRAF* V600E mutation in both MA-like and epithelial-predominant WT-like areas. Atypical MA is not a neglected subtype of MA, possessing an uncommon histological morphology and a higher Ki-67 index, but shares common features of imaging, immunophenotype and gene expression with typical MA, and patients usually have a good clinical outcome. Differentiating atypical MA from other renal tumors with epithelial components is important because of their totally different prognoses. Thus, since *BRAF* V600E gene is extremely infrequent or absent in non-MA renal tumors, its high mutation rate led to its application as a specific marker for MA.

## Data availability statement

The original contributions presented in the study are included in the article/supplementary material. Further inquiries can be directed to the corresponding author.

## Ethics statement

The studies involving human participants were reviewed and approved by the ethics committee of the West China Hospital of Sichuan University. Written informed consent to participate in this study was provided by the participants’ legal guardian/next of kin. Written informed consent was obtained from the individual(s), and minor(s)’ legal guardian/next of kin, for the publication of any potentially identifiable images or data included in this article.

## Author contributions

NC, conceptualization, methodology, and manuscript revision. XY, conceptualization, validation and writing of the original draft. XP, JT, and LZ, data curation and visualization. XZ, formal analysis. QZ, supervision. All authors listed have made substantial, direct, and intellectual contributions to the work.

## Funding

This study was supported by grants from the National Natural Science Foundation of China (NSFC 81872107, 81872108), Sichuan Province Science and Technology Support Program (2021YFS0114), and Fundamental Research Funds for the Central Universities (2022SCU12042).

## Acknowledgments

We thank the patients and their families for their support and understanding of this study and the pathologists and urologists of West China Hospital for providing the clinicopathological data of the patients.

## Conflict of interest

The authors declare that the research was conducted in the absence of any commercial or financial relationships that could be construed as a potential conflict of interest.

## Publisher’s note

All claims expressed in this article are solely those of the authors and do not necessarily represent those of their affiliated organizations, or those of the publisher, the editors and the reviewers. Any product that may be evaluated in this article, or claim that may be made by its manufacturer, is not guaranteed or endorsed by the publisher.
